# A cancer stem cell model as the point of origin of cancer-associated fibroblasts in tumor microenvironment

**DOI:** 10.1038/s41598-017-07144-5

**Published:** 2017-07-28

**Authors:** Neha Nair, Anna Sanchez Calle, Maram Hussein Zahra, Marta Prieto-Vila, Aung Ko Ko Oo, Laura Hurley, Arun Vaidyanath, Akimasa Seno, Junko Masuda, Yoshiaki Iwasaki, Hiromi Tanaka, Tomonari Kasai, Masaharu Seno

**Affiliations:** 1 Nano-biotechnology, Department of Medical Bioengineering, Okayama University, 3.1.1 Tsushima-Naka, Kita-ku Okayama, 700-8530 Japan; 2 Menoufia University, Faculty of Science, Chemistry Department, Shebin El-Koom, 32511 Egypt; 3 National Cancer Center Research Institute, 5-1-1, Tsukiji, Chuo-ku Tokyo, 104-0045 Japan; 4 Cancer Biology Graduate Program, School of Medicine, Wayne State University, 110E Warren Avenue, Suite 2215, Detroit, MI 48201 USA; 5 Department of Gastroenterology and Hepatology, Graduate School of Medicine, Okayama University, Okayama, 700-8558 Japan; 6 Department of Medical and Molecular Genetics, Indiana University School of Medicine, 975 W. Walnut Street, IB-130, Indianapolis, IN 46202 USA

## Abstract

Cancer-associated fibroblasts (CAFs) are one of the most prominent cell types in the stromal compartment of the tumor microenvironment. CAFs support multiple aspects of cancer progression, including tumor initiation, invasion, and metastasis. The heterogeneous nature of the stromal microenvironment is attributed to the multiple sources from which the cells in this compartment originate. The present study provides the first evidence that cancer stem cells (CSCs) are one of the key sources of CAFs in the tumor niche. We generated CSC-like cells by treating mouse induced pluripotent stem cells with conditioned medium from breast cancer cell lines. The resulting cell population expressed both CSC and pluripotency markers, and the sphere-forming CSC-like cells formed subcutaneous tumors in nude mice. Intriguingly, these CSC-like cells always formed heterogeneous populations surrounded by myofibroblast-like cells. Based on this observation, we hypothesized that CSCs could be the source of the CAFs that support tumor maintenance and survival. To address this hypothesis, we induced the differentiation of spheres and purified the myofibroblast-like cells. The resulting cells exhibited a CAF-like phenotype, suggesting that they had differentiated into the subpopulations of cells that support CSC self-renewal. These findings provide novel insights into the dynamic interplay between various microenvironmental factors and CAFs in the CSC niche.

## Introduction

The tumor microenvironment (TME) plays an indispensable role in the development and progression of cancer. The stromal compartment of the TME is comprised of a variety of cell types, including endothelial cells, fibroblasts, and immune cells, each possessing distinct yet complementary functions that support tumor architecture and maintenance^1^. Recent insights into the dynamic coevolution of mutated epithelial cells and the adjacent stromal compartment during cancer progression have prompted researchers to focus on the study of stromal cells. Stromal cells make up more than 80% of tumor volume in pancreatic and breast cancer and play a key role in the development and progression of cancer^2^.

Cancer-associated fibroblasts (CAFs) in the stromal compartment of the TME play a key role in tumorigenesis by mediating tumor growth, angiogenesis, inflammation, stromal remodeling, drug resistance, and metastasis. The multifunctional role of CAFs is attributed to their ability to mediate crosstalk between numerous signaling pathways by secreting essential factors and *via* the extracellular matrix. Recent studies indicate that CAFs have substantial clinical implications in disease staging and cancer recurrence. However, CAFs have not been fully characterized due to several limitations^3^. First, the origin of CAFs remains unclear. CAFs potentially originate from epithelial cells, mesenchymal stem cells, adipocytes, resident fibroblasts, and bone marrow stem cells^4^. The heterogeneous origin of CAFs accounts for their broad range of characteristics and molecular markers, a feature that makes it difficult to accurately distinguish CAF subpopulations from one another. Second, since CAFs have the innate ability to utilize the surrounding microenvironment to support their own growth therefore it is difficult to isolate and maintain them. Notably, the microenvironment that supports the growth of CAFs is similar to the microenvironment that supports the viability of cancer stem cells (CSCs). Recent studies suggest that several types of stromal cells in the CSC niche play pivotal roles in maintaining the small population of CSCs responsible for cancer recurrence and drug resistance^4^. However it is unclear if CSCs directly support tumor maintenance and survival by generating CAFs. Although there is evidence to support the hypothesis that CAF-mediated paracrine signaling preserves the stemness of patient-derived primary CSCs over time^5^, this hypothesis has yet to be confirmed. Our group recently developed a unique CSC model from mouse induced pluripotent stem (miPS) cells cultured with cancer cell-conditioned medium that mimicked the conditions of the tumor niche^6^. Using this model, we found that CSCs gave rise to vascular endothelial-like cells, thereby creating a niche that maintained the balance between self-renewal and differentiation, and supported the growth of heterogeneous tumors^7^. Furthermore, we generated a pancreatic ductal adenocarcinoma CSC model to study the effects of TME factors *in vivo* and a mechanism of CSC differentiation mediated by the maintenance of self-renewal potential and integrity^8^.

In the present study, we tested our hypothesis that CSCs can differentiate into CAF-like cells (CAFLCs) in the cancer niche. We generated CSCs by treating miPS cells with conditioned medium from BT549 or T47D cells, two breast cancer cell lines representing different hormone subtypes. The resulting CSC-like cells formed spheres that differentiated into various cell types, including myofibroblast-like cells. Further analysis revealed that the myofibroblast-like cells phenotypically resembled CAFLCs, supporting our hypothesis that CSCs might be a key source of CAFs in the tumor niche. Furthermore, our *in vitro* CSC model system provides a unique tool for analyzing the role of CAFs derived from CSC-like cells in the tumor microenvironment.

## Results

### miPS cells treated with breast cancer cell-conditioned medium differentiate into CSC-like cells

**Figure 1:**
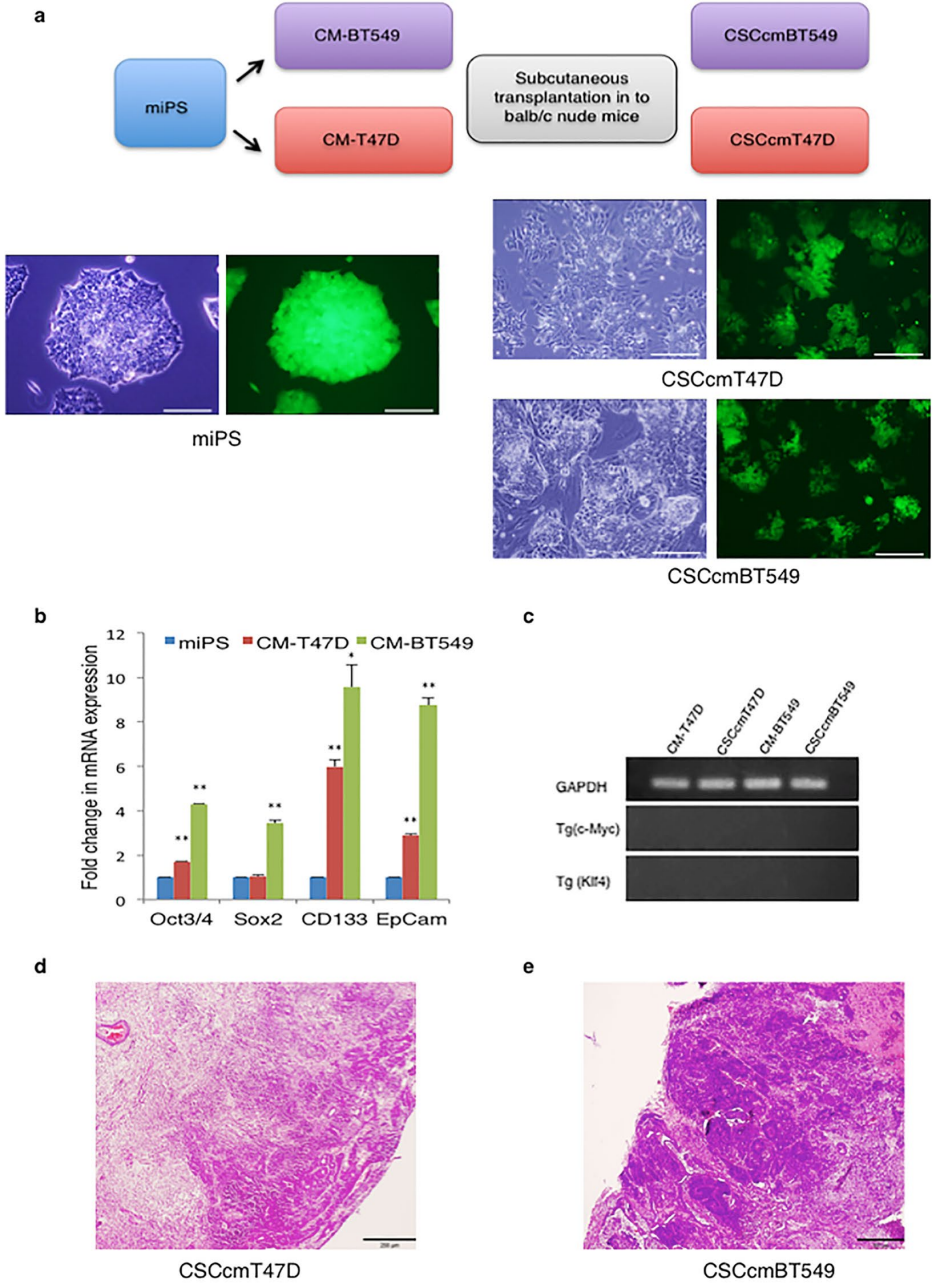
Differentiation of miPS cells into CSCs. (**a**) Schematic flow chart of CSC generation. Detailed description is in the text. Representative images of miPS cells, and CSCcmT47D and CSCcmBT549 cells. Images of unstained cells and GFP-positive cells from identical fields of view are shown side by side. Scale bars represent 200 μm. (**b**) RT-qPCR analysis of the CSC markers CD133, Epcam, Oct3/4, and Sox2 in CM-T47D and CM-BT549 cells after 4 weeks in CM. Gene expression levels were normalized to those of GAPDH, and relative gene expression levels in CM-T47D or CM-BT549 cells were compared with those in untreated miPS cells. (**c**) RT-qPCR analysis of the Klf4 and c-Myc transgenes in CM-T47D and CM-BT549 cells after four weeks in CM, and in primary CSCcmBT549 and CSCcmT47D cells generated from subcutaneous tumors. An image of an agarose gel with the RT-qPCR products demonstrates the absence of Klf4 and c-Myc expression. The data were analyzed using an unpaired two-tailed Student’s *t*-test and are presented as the mean ± standard deviation **P < 0.01, *P < 0.05. (**d** and **e**) T47DCSCcm (**d**) and BT549CSCcm (**e**) primary subcutaneous tumors stained with hematoxylin-eosin. Scale bars represent 300 μm.

Our group previously established a protocol to generate CSC-like cells by culturing miPS cells in conditioned medium from mouse cancer cell lines. Our findings suggested that cancer cell-conditioned medium is a rich source of secreted factors that potentially mimic the TME^6, 8^. In this study, we used miPS cells expressing a gene encoding green fluorescent protein (GFP) under the control of *Nanog* promoter, thereby allowing us to distinguish self-renewing undifferentiated CSCs from differentiated CSCs by the presence or absence of GFP expression, respectively. Conditioned medium was generated from two different breast cancer cell lines, T47D and BT549; respective culture medium was collected in 5% serum condition according to the previously established protocol^6^. Subsequently, the miPS cells were treated with this conditioned medium for a period of four weeks by changing the medium every second day. Untreated miPS cells failed to survive beyond one week because they require Leukemia Inhibitory Factor (LIF) and feeder cells to survive *in vitro* (Supplementary Fig. 1a). In contrast, miPS cells cultured in conditioned medium (CM-T47D and CM-BT549 cells) proliferated. CM-T47D and CM-BT549 cells were morphologically heterogeneous and consisted of a significant number of GFP-positive subpopulations (Fig. 1a and Supplementary Fig. 1b). To determine if CM-T47D and CM-BT549 cells maintained the expression of pluripotency markers, we analyzed gene expression in both GFP-positive and GFP-negative cells using quantitative reverse transcription PCR (RT-qPCR). CM-T47D and CM-BT549 cells expressed the pluripotency markers Oct3/4 and Sox2, whereas they did not express the Klf4 and c-Myc transgenes, suggesting that the identity of CM-T47D and CM-BT549 cells could primarily be controlled by undetermined endogenous genes (Fig. 1b,c). Notably, the key CSC marker CD133 and the epithelial carcinoma marker EpCam were expressed at levels 3- to 10-fold higher in CM-T47D and CM-BT549 cells compared with untreated miPS cells. Furthermore, the sustained expression of GFP supports the hypothesis that CM-T47D and CM-BT549 cells had differentiated into CSC-like cells (Fig. 1a) because *Nanog* reportedly promotes CSC-like characteristics^9^. These findings suggest that differentiation into CSC-like cells was induced by changes in endogenous gene expression mediated by factors secreted from the conditioned medium and that the conditioned medium mimics the TME. Next, we evaluated the tumorigenicity of the heterogeneous CSC-like cell population by subcutaneously transplanting the cells into Balb/c nude mice. The transplanted cells formed tumors with a high nuclear to cytoplasmic ratio and poorly-differentiated glandular structures characteristic of adenocarcinoma (Fig. 1a,d and e). Notably, we previously demonstrated that untreated miPS cells subcutaneously transplanted into nude mice only formed non-cancerous teratomas^6^. Flow cytometry analysis showed that conditioned medium-treated cells, CM-T47D and CM-BT549 were composed of a certain degree of CD44^+^/CD24^−/low^ expressing population, while its expression seems to be more pronounced in the primary culture cells (Supplementary Fig. 2). Subsequently, serial subcutaneous transplantations also gave rise to tumors, indicating that the cells were enriched for CSCs and that they maintained their tumorigenicity (Supplementary Fig. 3a). It is also suggested that human epidermal growth factor receptor (Her2) is a marker found to be associated with the CSC population in breast cancer tumors^10^. Therefore, we evaluated Her2 expression in tissue sections from orthotropic tumor transplants in the third mammary fat pad of Balb/c nude mice. Immunohistochemistry analysis of tumor tissue sections revealed high levels of Her2, indicating that breast CSCs population had become enriched *in vivo* (Supplementary Fig. 3b).

As the original miPS cells express a puromycin resistance gene, we used puromycin selection to completely deplete the tumors of cells originating from the nude mice. Following this selection process, the cells were renamed CSCcmT47D or CSCcmBT549 cells according to the conditioned medium in which they had originally been cultured. After the puromycin selection, primary CSCcmT47D and CSCcmBT549 cells exhibited a greater number GFP-expressing colonies surrounded by myofibroblast-like cells. This observation confirmed that miPS-derived CSC-like cells were present in the primary cultures (Fig. 1a). Since our flow cytometry analysis clearly indicated the enrichment of cells displaying markers associated with CSC in primary cultures and previous studies regarding the establishment of pancreatic ductal adenocarcinoma mouse model had demonstrated the same by RNA sequencing analysis^8^. Therefore, all further experiments were conducted using primary culture cells.

### Sphere-formation and differentiation are critical for a hierarchy of cancer stem progenitor cells

**Figure 2:**
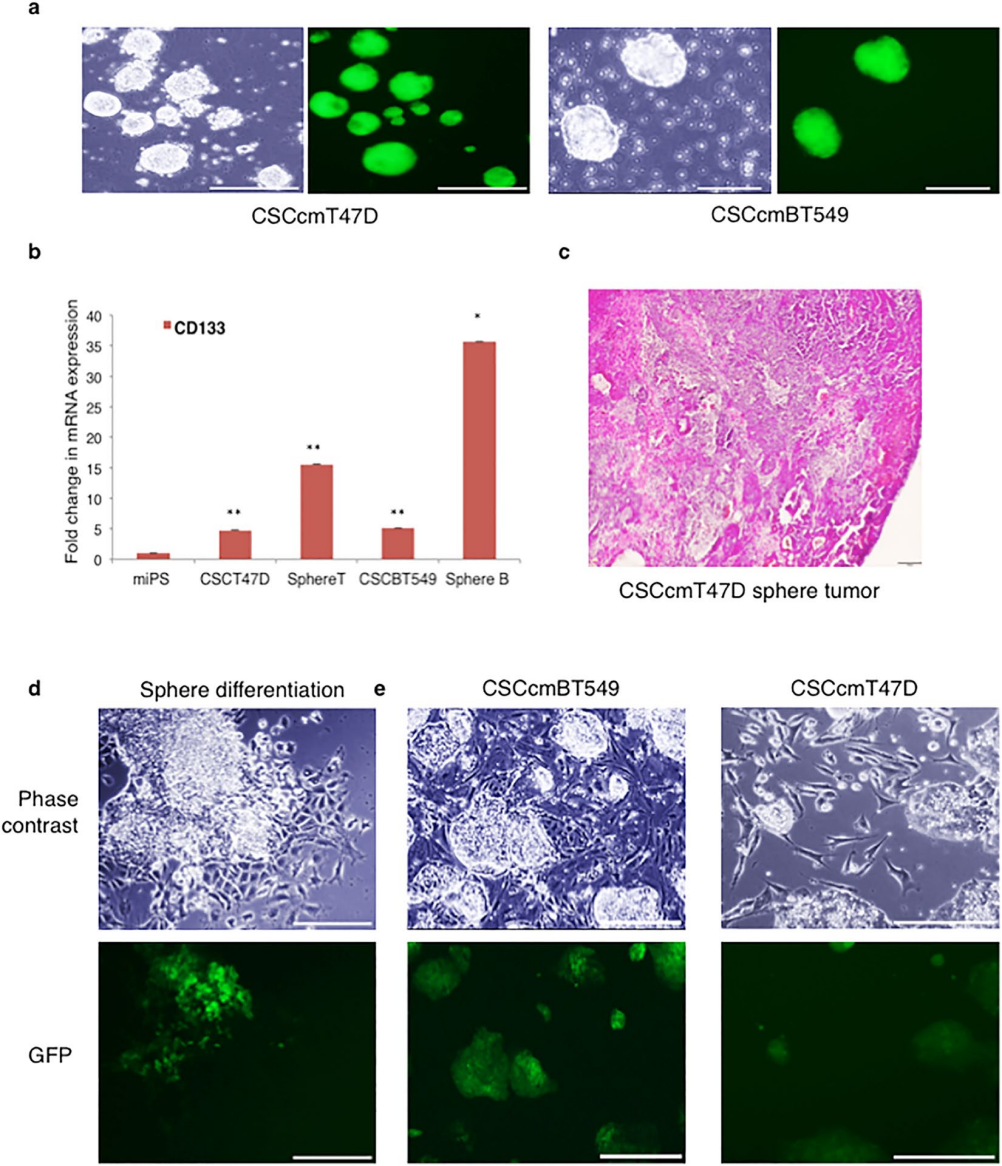
Sphere formation and CSC enrichment. (**a**) Representative images of sphere-forming cells. Images of unstained cells and GFP-labeled cells from identical fields of view are shown side by side. Sphere formation in tumor-derived primary CSCmT47D and CSCcmBT549 cells were cultured in serum-free conditions for 4 days. Scale bars represent 200 μm. (**b**) RT-qPCR analysis of CD133 expression in primary CSCcmT47D and CSCcmBT549 cells and their corresponding spheroids (sphereT and sphereB, respectively). Expression levels of the target genes were normalized to those of *GAPDH*, and untreated miPS cells were used as control cells. The data were analyzed using an unpaired two-tailed Student’s *t*-test and are presented as the mean ± standard deviation. **P < 0.01, *P < 0.05. (**c**) Histopathological analysis of primary subcutaneous tumors generated from injected CSCcmT47D spheroids. Scale bars represent 300 μm. (**d**) Morphology of primary cells generated from CSCcmBT549 spheroids. GFP expression was downregulated in CSCcmBT549 spheroids. The morphology of adherent CSCcmT47D spheroids was similar to that of CSCcmBT549 spheroids (data not shown). Scale bar represents 200 μm. (**e**) Representative morphology of cells derived from CSCcmT47D and CSCcmBT549 spheroids after two passages. Scale bars represent 200 μm and 400 μm.

Previous evidence suggests that the ability to form spheres is associated with CSC properties^11^. Therefore, we conducted sphere formation assays to further evaluate the CSC-like characteristics of CSCcmT47D and CSCcmBT549 cells. Sphere formation was significantly enhanced in CSCcmT47D and CSCcmBT549 cells compared to control cells (Fig. 2a), and single cells derived from dissociated spheres gave rise to new spheres over serial passages (Supplementary Fig. 4). RT-qPCR analysis revealed that the CSC marker CD133 was expressed at higher levels in spheres (sphereT and sphereB in Fig. 2b) compared with primary CSCcmT47D and CSCcmBT549 adherent cells, suggesting that the spheres were enriched for CSC-like cells (Fig. 2b). Sphere-forming cells subcutaneously transplanted into nude mice formed tumors, indicating that they retained their tumorigenicity (Fig. 2c). To determine the lineage of cells originating from the enriched CSC, the spheres were induced to differentiate under anchorage-dependent conditions. The cells were cultured in conditioned medium collected from CSCcmT47D or CSCcmBT549 cells that had been cultured in DMEM supplemented with 5% knockout serum replacement (KSR) (Fig. 2d). As differentiated cells did not survive in the original 1:1 conditioned medium, we assumed that undetermined factors in the primary cell-conditioned medium induced the spheres to differentiate. The differentiated cells consisted of heterogeneous sphere-like colonies of GFP-expressing cells surrounded by myofibroblast-like cells. Interestingly, even after multiple rounds of dissociation and passaging, both undifferentiated and differentiated cells were observed. Notably, the myofibroblast-like cells directly surrounded the sphere-like cells. Based on these observations, we hypothesized that the close interaction between these subpopulations might be required for CSC self-renewal and maintenance (Fig. 2e)^7^.

### Myofibroblast-like cells from CSC colonies exhibit a CAF-like phenotype

**Figure 3:**
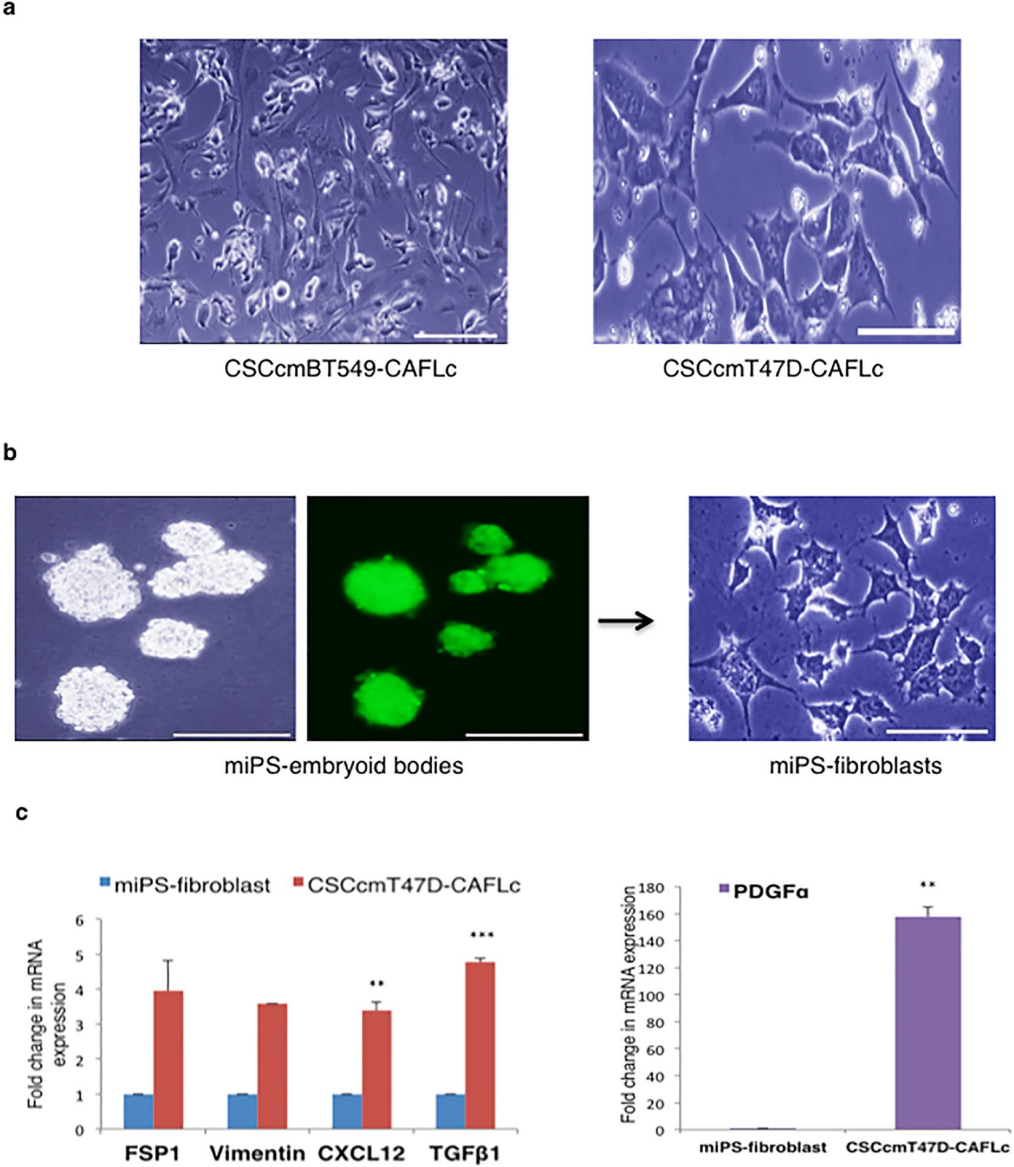
Characterization of differentiating CAFLCs. Morphology of differentiated cells isolated from primary CSCcmT47D and CSCcmBT549 spheroids. The cells exhibited prominent nucleoli with long spindles. Scale bar represents 200 μm (*right*) and 100 μm (*left*). (**b**) Morphology of fibroblasts derived from miPS embryoid bodies. Scale bar represents 200 μm. (**c**) RT-qPCR analysis of the CAF markers FSP1, vimentin, TGFβ1, CXCL12, Col1α1, and PDGFα in CSCcmT47D-CAFLCs. The data were analyzed using an unpaired two-tailed Student’s *t*-test and are presented as the mean ± standard deviation. ***P < 0.001, **P < 0.01, *P < 0.05.

**Figure 4:**
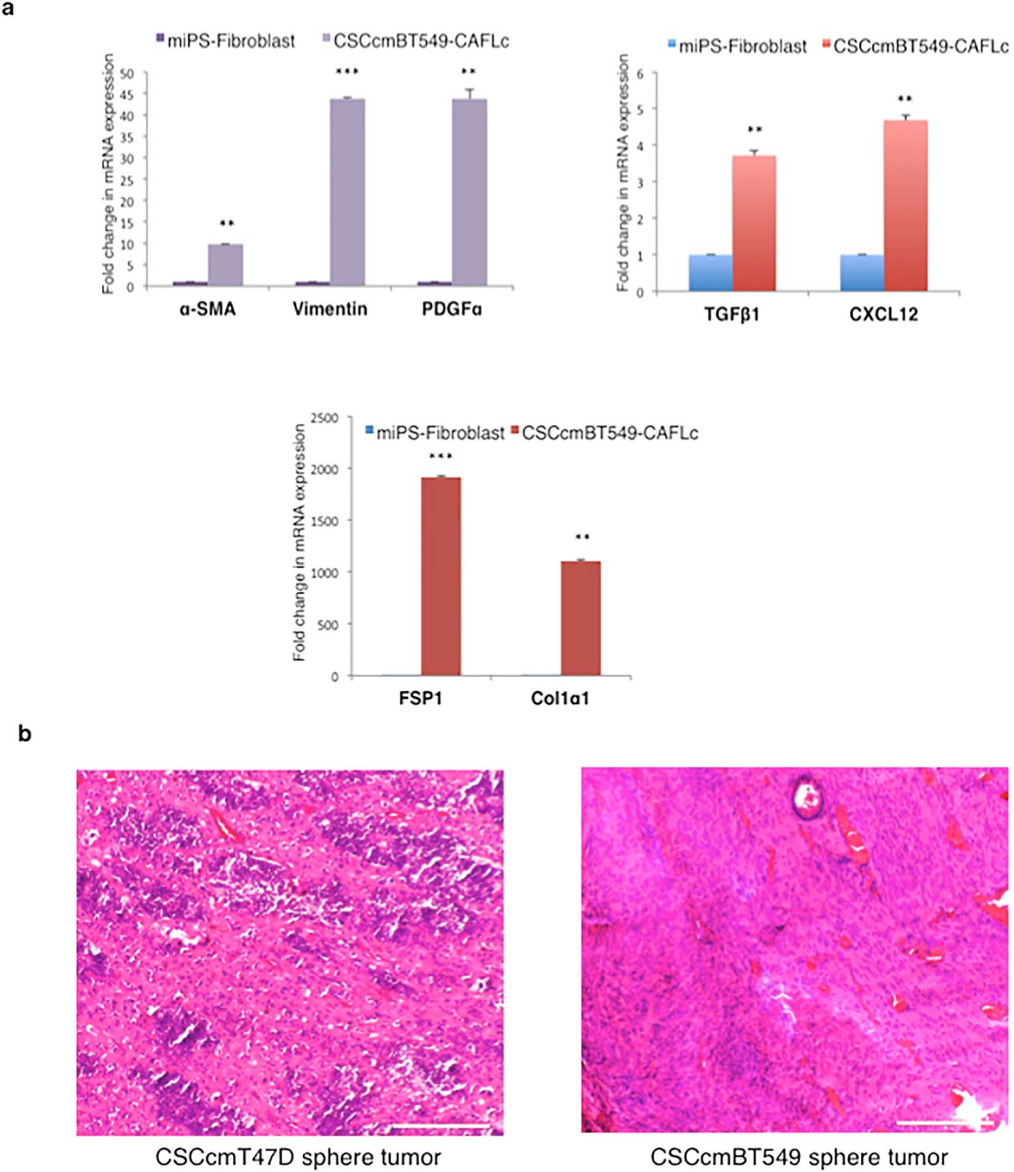
Characterization of differentiating CAFLCs and histochemistry. (**a**) RT-qPCR analysis of the CAF markers FSP1, vimentin, TGFβ1, CXCL12, and PDGFα in CSCcmBT549-CAFLCs. The data were analyzed using an unpaired two-tailed Student’s *t*-test and are presented as the mean ± standard deviation. ***P < 0.001, **P < 0.01, *P < 0.05. (**b**) Hematoxylin-eosin staining of subcutaneous tumors generated from CSCcmT47D and CSCcmBT549 spheres. Scale bars represent 200 μm.

Although multiple origins of CAFs have been proposed in recent studies, the origin of TME heterogeneity remains elusive^12^. To investigate the role of CAFs in the TME, we separated differentiated myofibroblast-like cells from sphere-derived CSCs using mouse feeder-specific magnetic microbeads or magnetic activated cell sorting (MACS) (Fig. 3a). To generate control cells, the original miPS cells were induced to differentiate into fibroblast-like cells using previously reported protocols^13, 14^. After the cells differentiated, they were separated using magnetic beads. The pure fibroblasts isolated from the original miPS cells are referred to as miPS-fibroblasts (Fig. 3b). We quantitatively analyzed the expression of the CAF markers fibroblast specific protein (FSP1), α-smooth muscle actin (α-SMA), stromal derived factor-1 (CXCL12), and transforming growth factor (TGFβ1), as well as platelet derived growth factor (PDGFα), alpha-1 type collagen (Col1α1), and the fibroblast-specific marker vimentin. All of these genes were upregulated in myofibroblast-like cells compared with miPS-fibroblasts (Figs 3c and 4a). These findings supported our hypothesis that differentiated myofibroblast-like cells from sphere-derived CSCs resemble CAFs. Therefore, we named these cells CSCcmT47D-CAFLCs or CSCcmBT549-CAFLCs. A recent study of the TME reported that tumors are composed of various subpopulations of CAFs^15^. Consistent with these findings, spheres generated from primary CSCcmT47D and CSCcmBT549 cells differentiated into a heterogeneous population of CAFs (CSCcmT47D-CAFLCs or CSCcmBT549-CAFLCs). Compared with miPS-fibroblasts, CSCcmT47D-CAFLCs expressed higher levels of α-SMA, CXCL12, TGFβ1, Vimentin, and PDGFα; whereas, α-SMA expression was undetectable (Fig. 3c and data not shown). In contrast, CSCcmBT549-CAFLCs expressed high levels of genes encoding CAF secretory factors, as well as α-SMA, Vimentin, and Col1α1 (Fig. 4a). Moreover, hematoxylin and eosin (H&E) staining revealed that CSCcmT47D and CSCcmBT549 spheroid cell-derived tumors were highly enriched for stromal cells (Fig. 4b), consistent with the results of our *in vitro* experiments (Figs 3c and 4a). These observations further confirm that breast cancer cell-conditioned medium plays a pivotal role in the differentiation of CSC spheres.

### CSC-derived CAFs express CAF markers and have high invasive potential

**Figure 5:**
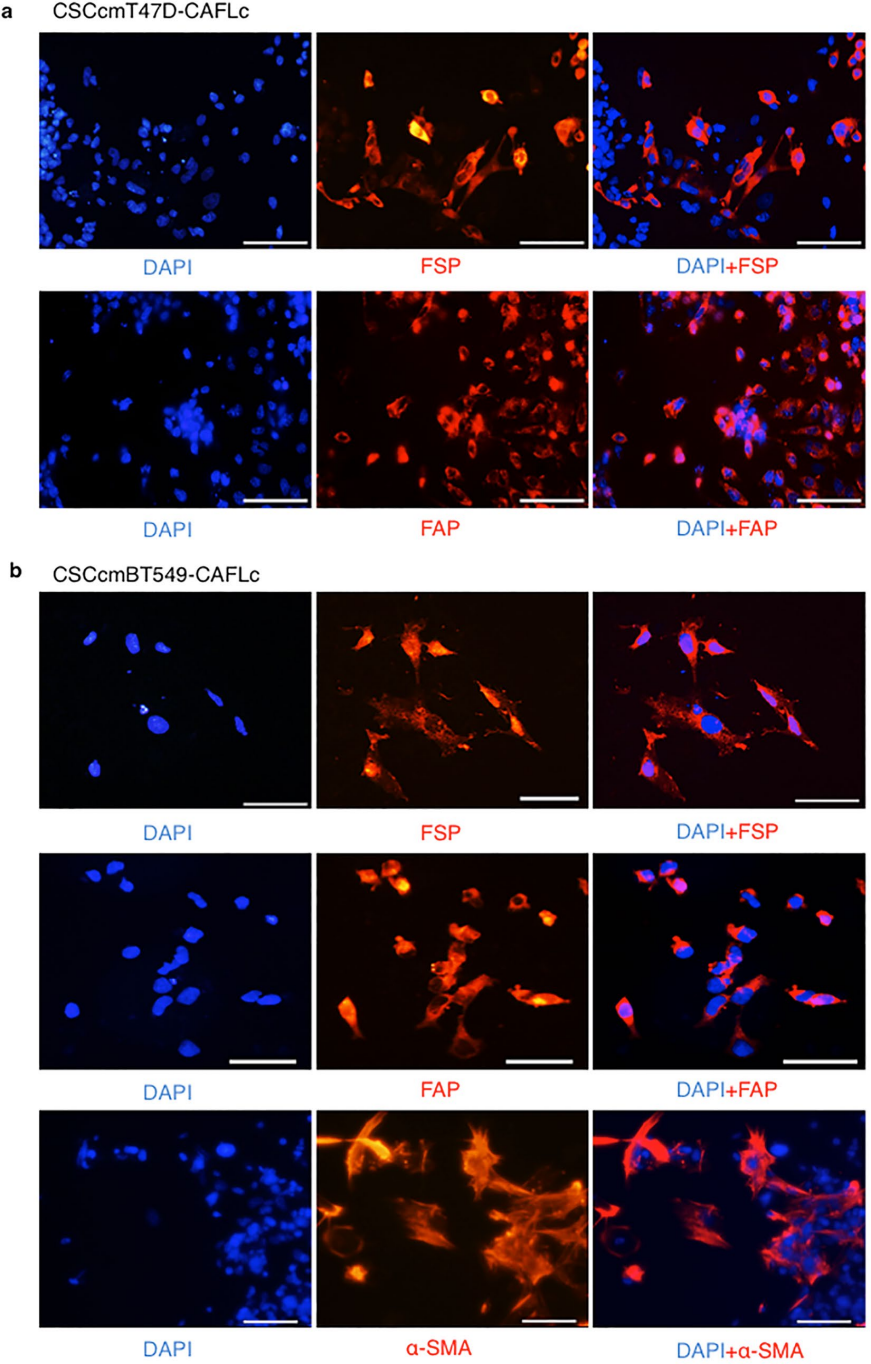
Immunofluorescence analysis of CAF markers. Isolated CSCcmT47D-CAFLCs (**a**) and CSCcmBT549-CAFLCs (**b**) were stained with anti-rabbit FSP1, anti-rabbit α-SMA, or anti-rabbit FAP (*red*). The nuclei were counterstained with DAPI (*blue*). Scale bars represent 50 μm.

**Figure 6:**
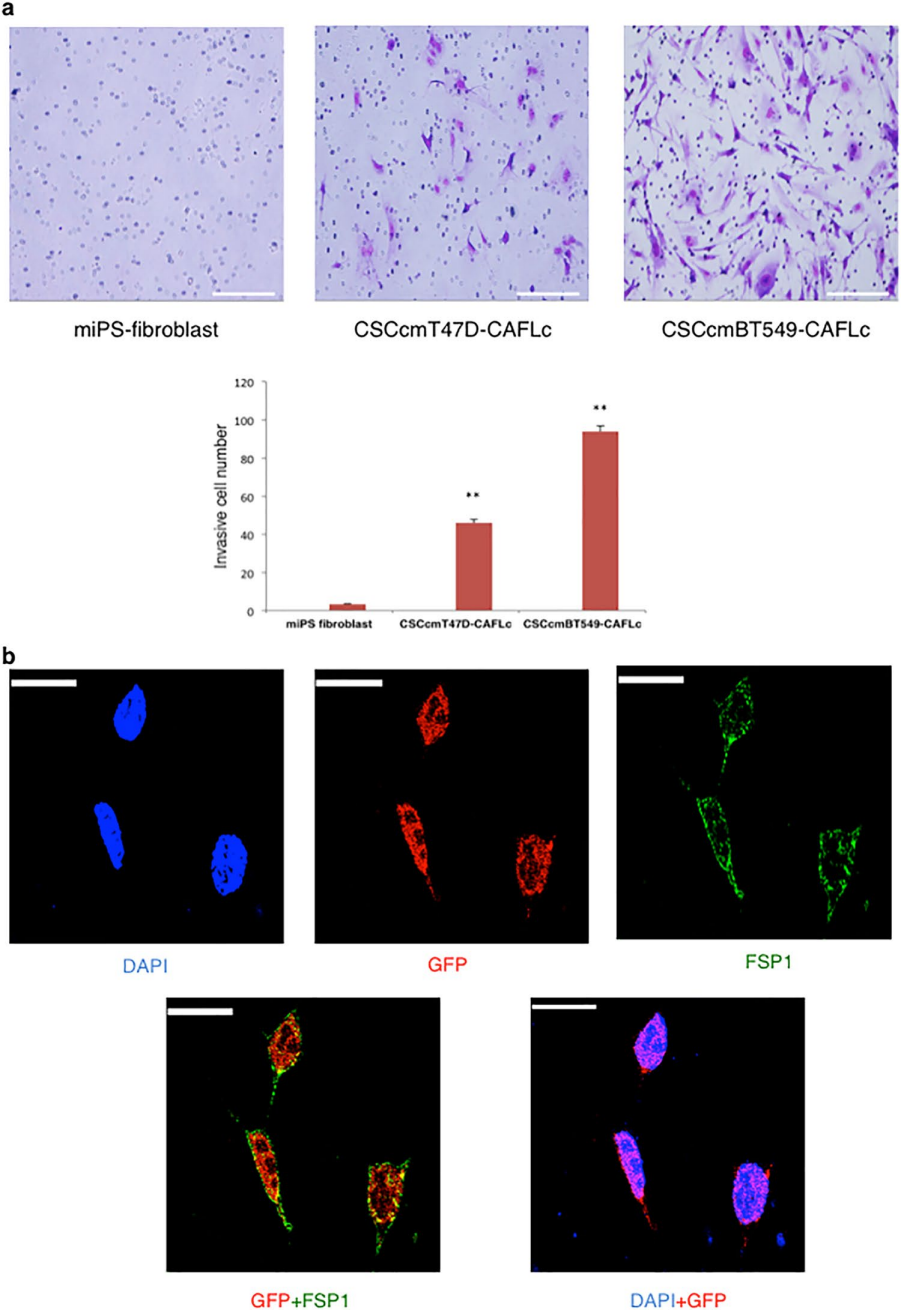
Assessing enhanced invasion potential and lineage tracing. (**a**) CAFLC invasion ability was measured using Matrigel-coated transverse inserts. Invasive cells were stained with Giemsa and quantitatively analyzed in several fields of view. The experiments were conducted in triplicate. Scale bar represents 200 μm. The graph indicates quantitative results by counting invasive cells. CSCcmT47D-CAFLCs and CSCcmBT549-CAFLCs were more invasive than control miPS-fibroblasts. The data were analyzed using an unpaired two-tailed Student’s *t*-test and are presented as the mean ± standard deviation. **P < 0.01, *P < 0.05. (**b**) *In situ* hybridization analysis using a probe targeting GFP (*red*) and immunofluorescence analysis of FSP1 (green). The strong GFP signal in the nucleus indicated that CAFs retained plasticity. Scale bar represents 10 μm.

Recent studies characterizing CAFs used multiple markers, as there is no single distinguishing marker associated with CAFs. We chose to analyze the CAF markers FSP1, α-SMA, and fibroblast activation protein alpha (FAP) using immunohistochemistry, because these proteins are considered key markers for identifying major subpopulations of CAF^4^. CSCcmBT549-CAFLCs expressed all three markers, whereas CSCcmT47D-CAFLCs expressed FSP1 and FAP, but not α-SMA (Fig. 5a,b). These findings were consistent with the results of RT-qPCR assays (Figs 3c and 4a). Therefore, we propose that the respective CSC-conditioned medium could contribute significantly to the differentiation from CSCs to CAFs. A previous study using GFP-labelled CAFs demonstrated that CAFs might guide tumor cell migration at the invasive front^16^. Using a Matrigel invasion assay, we confirmed that CSCcmT47D-CAFLCs and CSCcm BT549-CAFLCs were highly invasive compared with miPS-fibroblasts (Fig. 6a). To trace the lineage of CSCcmT47D-CAFLCs, we conducted *in situ* hybridization for GFP in combination with immunofluorescence staining for FSP1. Unexpectedly, the GFP and FSP1 signals were significantly stronger in the nucleus compared with the cytoplasm (Fig. 6b). In contrast, GFP florescence intensity in primary CAFLCs was attenuated. We speculate that, although CSCs differentiate into CAFLCs, they could be multipotent and maintain phenotypic plasticity. A previous study reported that multipotent primary CAFs were present in hepatocellular carcinoma tissue^16^. Another study reported that CAFs isolated from colon cancer patients retained a stem cell-like phenotype^17^. These previous reports reinforce our findings that CAFLCs originate from CSC-like cells.

## Discussion

The present study provides the first experimental evidence suggesting that CSCs are a key source of CAFs in the TME. We generated CSCs by treating miPS cells with conditioned medium from BT549 (claudin low) or T47D (luminal A) breast cancer cells. These cell lines were initially established from invasive ductal breast carcinoma. We chose to use cancer cell-conditioned medium to evaluate the effects of diverse tumor niche-derived factors on CSC differentiation in order to evade the caveats associated with introducing genetic modifications. We found that GFP-positive cells treated with conditioned medium upregulated the expression of CD133 and and the epithelial carcinoma marker found to overexpressed in breast carcinoma Epcam in addition to CD44^+^/CD24^−/low^, suggesting that they had differentiated into CSC-like cells. Indeed, previous studies from several groups have demonstrated that conditioned medium from non-cancerous cell type did not support the generation of cells with oncogenic potential, although this medium was inducing differentiation of the pluripotent stem/iPS cells^18–21^. Therefore, it is unlikely that the conditioned medium from non-cancerous cells is capable of converting iPS cells into cancer stem cell state. The CM-T47D and CM-BT549 cells were morphologically heterogeneous; therefore, we conducted subcutaneous transplantation experiments to enrich the population of CSC-like cells. *In vivo* transplantation is a valuable approach for evaluating the tumorigenic potential of CSCs, as the tumor microenvironment facilitates CSC enrichment^22^. Cells treated with the conditioned medium formed tumors with a high nuclear to cytoplasmic ratio and glandular structures indicative of adenocarcinoma-like phenotype. Moreover, subsequent serial transplantations did not attenuate their tumorigenicity. Therefore, this approach facilitated the study of CSC lineage *in vitro*. In addition, the enrichment of CSCs *in vivo* allowed us to evade the limitations associated with CSCs isolated from cancer cell lines^7^.

Sphere formation is a key indicator of CSC tumorigenicity^11^. The CSCs we established gave rise to a significant number of spheres in non-adherent serum-free conditions. GFP expression facilitated the selection of self-renewing spheres from aggregates. Sphere-forming CSCs are considered more tumorigenic than their adherent counterparts^18^. Accordingly, spheres that were subcutaneously transplanted into nude mice formed tumors with adenocarcinoma-like features, thereby confirming their tumorigenic potential. Expression levels of CD133, a gene essential to CSC enrichment, increase 3- to 7-fold in CSC spheres^23^.

Under adherent conditions, spheres have the distinctive ability to differentiate into various cell types and to proliferate^24^. Notably, during this differentiation, feeder-like myofibroblast cells surrounded undifferentiated cell populations. We induced differentiation of spheres with primary CSCcmBT549- and CSCcmT47D-conditioned medium in which FBS was replaced with a low concentration of KSR medium diluted in DMEM. Many recent studies have preferentially used serum-free medium to avoid the potential for spontaneous and uncontrolled differentiation. The serum-free primary CSC-conditioned medium facilitated slower and more controlled differentiation in adherent conditions, thereby enabling us to evaluate the cell types that play an important role in maintaining the CSC population. Differentiated myofibroblast-like cells exhibited a spindle-shaped morphology with prominent nucleoli, and they expressed elevated levels of the CAF markers α-SMA, Vimentin. To confirm that this cell population did not include normal or activated fibroblasts, we compared them with miPS-fibroblasts. miPS-fibroblasts cells did not express CAF markers, indicating that CAFLCs originated from CSC-derived fibroblasts. Moreover, genes encoding CAF secretory factors that promote cancer cell invasion and disease progression, including CXCL12, TGFβ1, and PDGFα, were upregulated in CSC-derived fibroblasts. Consistent with these findings, immunohistochemistry assays demonstrated that CSC-derived fibroblast cells expressed the CAF markers FSP, α-SMA, and FAP. However, these markers were upregulated at the mRNA level but not the protein level. This discrepancy could be due to limitations associated with a lack of paracrine signaling and an inability to mimic the tumor niche *in vitro*. CAFs exhibited strong invasive potential compared with miPS-fibroblasts, consistent with the results of a previous study in which CAFs isolated from patient samples were more invasive then tumor cells^25^.

We also characterized the distinct subpopulations of CSC-derived CAFLCs. Zeisberg *et al*. previously reported that different types of CAFs express different markers and that the stromal compartment of the TME has distinct subpopulations of CAFs that exclusively express either FSP1 or α-SMA along with several other classes of unknown markers^26^. Our results indicate that CAF heterogeneity might result from the differences in epigenetic and TME changes associated with their CSC progenitors. Consistent with a previous report, we could not maintain CSC-derived CAFs for more than five passages^27^. Notably, some isolated CAFs subpopulations were able to revert to a stem cell-like state, and fully differentiated CSC-derived CAFs did not survive. This phenomenon was not observed in normal miPS cells. Ishikawa *et al*. demonstrated that primary cultures of patient-derived breast cancer cells exhibited both CAF and CSC-like properties^28^. Similarly, primary CSCcmBT549 and CSCcmT47D cells that gave rise to GFP-positive stem cell colonies surrounded by differentiated fibroblast-like cells survived repeated passages. Madar *et al*. proposed that the CAF is not a cell but a “state”. Consistent with this hypothesis, CAFs can switch between a stem cell-like state and a fully differentiated CAF-like state, thereby providing a niche that supports CSC survival^1^. A recent study demonstrated that primary cultures of patient-derived lung CSCs could be maintained for extended periods of time when CAFs were used as feeder cells^5^. This finding supports our hypothesis that CSCs require CAFs to survive.

To determine if our findings could be reproduced in other stem cell types, we conducted similar experiments using mouse embryonic stem (mES) cells treated with conditioned medium from BT549 cells to generate iPS-CSCs as previously described^6^. As these cells constitutively expressed GFP, we could continuously trace them as they differentiated into CAFs from CSC-like cells. The resulting CSC-like cells were subcutaneously injected into nude mice, and primary cells were cultured from the resulting tumors. Interestingly, the majority of the tumor stromal cells originated from the injected CSC-like cells (Supplementary Fig. 5). Cells expressing fibroblast-specific markers were isolated from the primary cell cultures using MACS and subsequently analyzed using immunohiostochemistry assays for the CAF markers FSP1, FAP, and α-SMA (Supplementary Fig. 6). CSC-like cells that gave rise to CAFLCs were identified by the coexpression of GFP and CAF markers. To further verify that CSC-like cells could differentiate into CAFLCs, we evaluated spheres generated from BT549 cells. Spheres collected from cells cultured in serum-free conditions were allowed to differentiate in adherent conditions in the presence of BT549-conditioned medium. Some differentiated cells expressed the CAF markers FSP1 and α-SMA (Supplementary Fig. 7). Together, these observations support our hypothesis that CSCs are a potential source of CAFLCs.

The present study demonstrated that CAFs in the TME play an indispensable role in the maintenance of CSCs. There are several reports that CAFs and/or CAF-conditioned medium contribute to cancer cell metastasis by promoting the epithelial-to-mesenchymal transition^15^. Although the precise mechanism by which CSCs differentiate into CAFs needs to be deciphered, we generated preliminary evidence that TGFβ1 plays a role in this process. CSCcmT47D spheres cultured in serum-free differentiation medium supplemented with 10 ng/ml TGFβ1 differentiated into CAFLCs that express FSP1, Vimentin, Col1α1, and CXCL12. Similar to spheres cultured in conditioned medium, these cells did not express α-SMA. However, the CAFLCs could not be maintained for more than two passages, and they did not form GFP-positive CSC colonies surrounded by fibroblast-like cells. These findings indicate that additional niche factors and paracrine signaling are required for this phenomenon (Supplementary Fig. 8).

In summary, we demonstrated that CSCs are a key source of CAFLCs in the TME. CAFLCs are dependent on one other for survival. The heterogeneity of CAFs in different types of cancer could be explained by understanding the mechanism of CSC-to-CAF conversions. We propose that inhibiting the conversion of CSCs to CAFs might have potential therapeutic implications in the future.

## Materials and Methods

### Cell culture

The human breast cancer cell lines, T47D (ATCC HTB-133) and BT549 (ATCC HTB-122), were cultured in RPMI-1640 (Sigma) medium supplemented with 10% fetal bovine serum (FBS) (Gibco, NY) and 100 U/ml penicillin/streptomycin (P/S) (Wako, Japan). The conditioned medium (CM) was collected as previously described^6^. miPS (iPS MEF-Ng-20D-17) cells (Riken Cell Bank, Japan)^29^ were maintained in DMEM (Sigma) supplemented with 15% FBS, 0.1 mM MEM non-essential amino acids (NEAA) (Gibco), 2 mM L-glutamine (Nacalai Tesque, Japan), 50 U/ml P/S, 0.1 mM 2-mercaptoethanol (Millipore, MA), and 1000 U/ml Leukemia Inhibitory Factor (LIF) (Millipore, MA) on a feeder layer of mitomycin-treated mouse embryonic fibroblast (MEF) cells (Reprocell, Japan). The miPS cells expressed *GFP* under the control of the *Nanog* promoter. For the conditioned medium treatment, miPS cells were cultured with CM and miPS medium (1:1) in the absence of LIF and MEF feeder cells, and the medium was changed every 24 hours for 4 weeks. Primary CSCs derived from mouse allografts was maintained in the same medium. To induce sphere formation, primary CSCs were cultured in miPS medium supplemented with 10% FBS and 50 U/ml P/S until they reached 80% cell confluence. Then, the medium was replaced with DMEM supplemented with 5% knockout serum replacement (KSR) (Gibco), 0.1 mM NEAA, 2 mM glutamine, and 50 U/ml P/S. The medium was collected after the cells reached 100% confluence. The CM was centrifuged at 1000 rpm for 5 minutes, purified through a filter with 0.22 μm-diameter pores (Millipore, Ireland), and stored at −20 °C until further use.

### Sphere formation, differentiation, and separation

The cells (5 × 10^4^) were seeded on 6-cm ultra-low attachment dishes (Corning Incorporated, NY) in FBS-free DMEM supplemented with Insulin-Transferrin-Selenium-X (ITS-X) (Life Technologies, CA), 0.1 mM NEAA, 2 mM L-glutamine, and P/S. After 3–4 days, sphere-forming cells were collected by centrifuging the cells at 300 rpm for 5 minutes and resuspending them in 1× phosphate buffered saline (PBS). After aspirating the PBS, the spheres were dissociated using Accutase® cell detachment solution (Sigma, USA). Then, the cells were seeded at a density of 1 × 10^4^ cells/ml in ultra-low attachment dishes the same medium. After 4 days, spheres with a diameter of approximately 100 μm were seeded in 6-cm adherent dishes coated with 0.1% gelatin (Sigma) containing a 1:1 ratio of DMEM supplemented with 5% KSR and P/S, and CM from the primary CSCs. The medium was changed every other day, and differentiated cells were maintained for no longer than 2–3 passages before being dissociated. Differentiated cells were separated using feeder removal microbeads, an LS column, and a Midi MACS separator (Miltenyi Biotec, Singapore). The cells were subsequently maintained in the same medium supplemented with KSR for up to 5 passages.

### Animal experiments

Four-week-old female Balb/c-nu/nu mice (Charles River, Japan) were subcutaneously injected with 1 × 10^6^ cells suspended in 200 μl of Hanks Balanced Salt Solution (HBSS) (Gibco). Tumors were harvested once they grew to a size of approximately 1000 mm^3^. Primary spheres were resuspended in 200 μl of Matrigel matrix (Corning Incorporated, NY) and subcutaneously injected into 4-week-old nude mice. The protocol for the animal experiments was reviewed and approved by the Animal Care and Use Committee of Okayama University under ID OKU2013252, OKU2014157, OKU2014429, and OKU2016078. All of the experiments were conducted in accordance with the Policy on the Care and Use of Laboratory Animals, Okayama University.

### Histological analysis

Tumors were excised from the mice 4 weeks post-transplantation. The tumors were fixed in 4% paraformaldehye (Wako, Japan) and embedded in paraffin. The paraffin tumor blocks were sectioned into 5 μm thick slices. Then, the sections were deparaffinized and stained with 0.5% hematoxylin (Sigma Aldrich) and Eosin Y (Sigma Aldrich) for histological analysis.

### Immunofluorescent staining

The cells were seeded at a density of 1 × 10^5^ cells/ml on 0.1% gelatin-coated coverslips in 24-well culture plates. The cells were fixed in 4% paraformaldehye for 20 minutes at room temperature, and subsequently permeabilized using 0.2% Triton-X (Nacalai Tesque, Japan) in PBS for 5 minutes. Then, the cells were washed and incubated in blocking solution (PBS supplemented with 5% BSA and FBS) for 1 hour. The cells were incubated overnight at 4 °C with the following primary antibodies: anti-rabbit FSP1 (1:300) (Merck Millipore, Ireland), anti-rabbit α-SMA (1:200) (Abcam), and anti-rabbit FAP alpha (1:200) (Abcam). After being washed with PBS, the cells were incubated with Alexa Fluor 555 donkey anti-rabbit IgG (1:300) (Invitrogen, USA) for 1 hour. Nuclei were counterstained with 4′,6-diamino-3-phenylidole, dihydrochloride (DAPI) (Sigma). The cells were mounted on glass sides using Vectashield mounting medium (Vector Labs, USA). Images were acquired using an Olympus microscope equipped with a fluorescence lamp (Olympus, Japan).

### RNA extraction and quantitative reverse transcription PCR (RT-qPCR)

Total RNA was extracted from cells using an RNAeasy Mini kit (QIAGEN, Germany), and the extracted RNA was treated with DNase I (Invitrogen, USA). One μg of RNA was reverse transcribed using a Superscript First-Strand kit (Invitrogen). qPCR assays were conducted using LightCycler 480 SYBR green I Master Mix (Roche, Switzerland) according to the manufacturer’s instructions. The primers used for the qPCR assays are described in Supplementary Table 6.

### Cell invasion assay

Cell invasion potential was evaluated using a Corning Matrigel Invasion Chamber, which consisted of a Matrigel-coated Transwell and Transwell inserts (Corning). The cells (5 × 10^4^ cells in 500 μl of DMEM) were added to the upper chamber, and medium supplemented with 15% FBS was added to the lower chamber. The cells were allowed to invade for 72 hours. Non-invasive cells were removed by wiping, and cells that had invaded the Matrigel were fixed in 4% paraformaldehye for 5 minutes and subsequently fixed in methanol (Wako, Japan) for 20 minutes. The cells were stained with Azure EMB Giemsa (Merck Millipore) and quantitatively analyzed under a light microscope at a magnification of 10x.

### Combined fluorescence *in situ* hybridization (FISH) and immunofluorescence

Combined FISH and immunofluorescence assays were conducted according to the manufacturers’ protocols. A Stellaris FISH probe (GFP labeled with Quasar 570 dye) “VSMF-1018-5” Biosearch Technologies, Inc., Petaluma, CA) was used for the FISH assays. Anti-FSP1 (1:300) (Merck Millipore) was used for the immunofluorescence assays.

### Flow cytometry

To isolate CD44^+^/CD24^low^ cells, primary cells derived from 3 independent tumor samples were separately stained with monoclonal anti-CD44 (Miltenyi Biotec), monoclonal anti-CD24 (CD24-PE: Miltenyi Biotec), and isotype controls (Rat IgG2b-APC and Rat IgG2b-PE) (Miltenyi Biotec) for 30 minutes on ice. The stained cells were analyzed using an Accuri™ flow cytometer (BD Biosciences) and FlowJo Software (Treestar Inc., San Carlos, CA).

## Electronic supplementary material

Supplementary Information
