# Supplementary material for: A cancer stem cell model as the point of origin of cancer-associated fibroblasts in tumor microenvironment
Source: Sci Rep. 2017 Jul 28;7:6838. doi: 10.1038/s41598-017-07144-5 (PMC5533745; doi:10.1038/s41598-017-07144-5)
Supplement: Supplementary file 1 — Supplementary Information [file 41598_2017_7144_MOESM1_ESM.pdf]

A cancer stem cell model as the point of origin of cancer-associated fibroblasts in tumor microenvironment

Neha Nair[1], Anna Sanchez Calle[1], Maram Hussein Zahra[2], Marta Preito-Villa[3], Aung Ko Ko Oo[1], Laura Hurley[4], Arun Vaidyanath[1], Akimasa Seno[1], Junko Masuda[1], Yoshiaki Iwasaki[5], Hiromi Tanaka[6], Tomonari Kasai[1] and Masaharu Seno[1]

1. Nano-biotechnology, Department of Medical Bioengineering, Okayama University, 3.1.1 Tsushima-Naka, Kita-ku, Okayama, 700-8350 Japan
2. Menoufia University, Faculty of Science, Chemistry Department, Shebib El-Koam, 32511 Egypt
3. National Cancer Research Centre, 5-1-1, Tsukiji, Chuo-ku, Tokyo, 104-0045 Japan
4. Cancer Biology Graduate Program, School of Medicine, Wayne State University, 10E warren Avenue, Suite 2215 Detroit, MI, 48201 USA
5. Department of Gastroenterology and Hepatology, Okayama University, 700-8558 Japan
6. Department of Medical and Molecular Genetics, Indiana University School of Medicine, 975 W. Walnut Street, IB-130, Indianapolis, IN, 460202 USA

Supplementary Figure 1

a

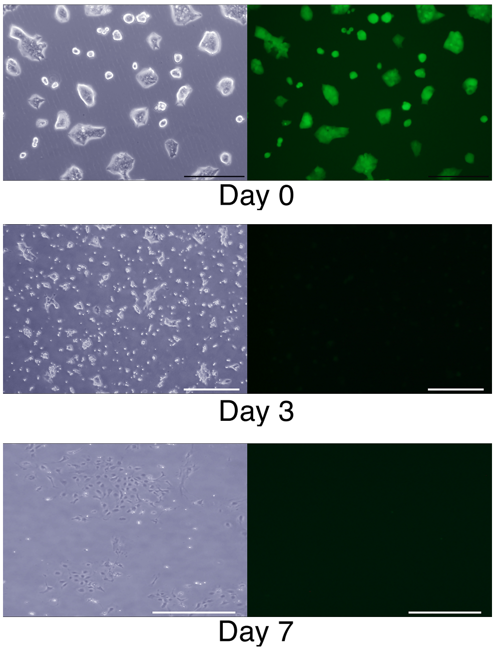

b

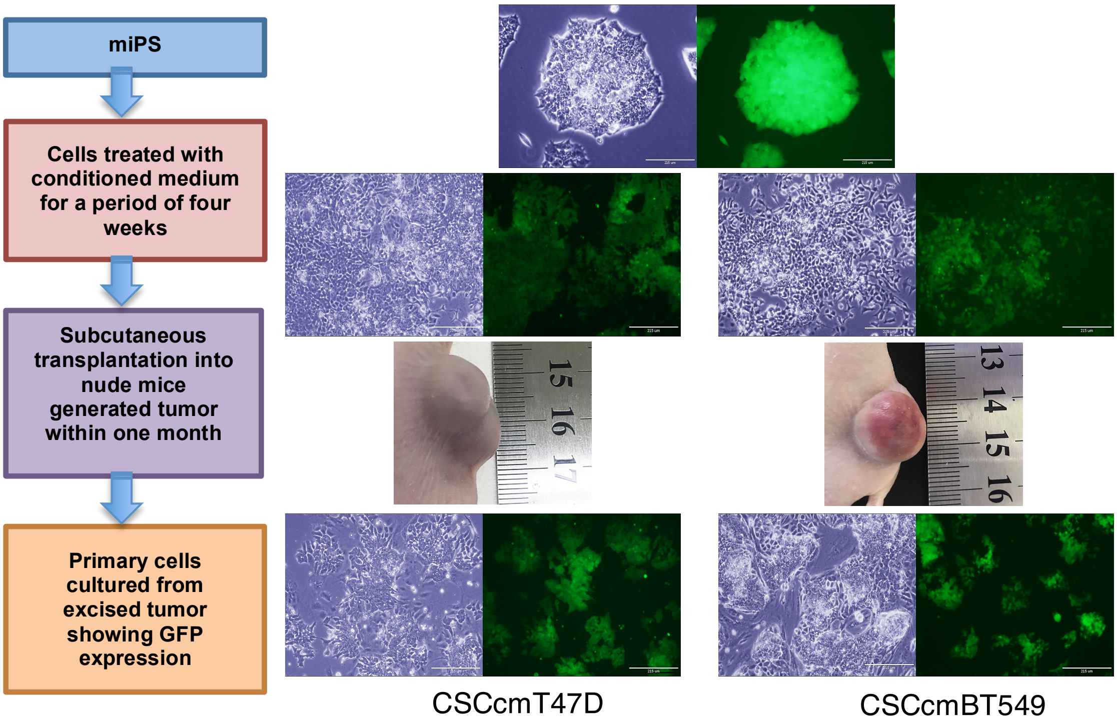

Supplementary Fig 1: (a) Representative images of miPS cells in feeder-less condition. Cells could not maintained without LIF for more than a week and expire with concomitant loss of GFP expression. Scale bars represent 200µm (day 0) and 400µm (day 3&7) (b) Schematic representation of the CSC establishment from miPS cells using the conditioned medium (CM) from T47D and BT549 cell lines. CSCcmT47D and CSCcmBT549 represent primary cells excising tumor from nude mice. Scale bars represent 200 µm. Unstained cells in phase contrast and GFP-labeled cells in fluorescence contrast from each identical viewfield are shown side by side.

Supplementary figure 2

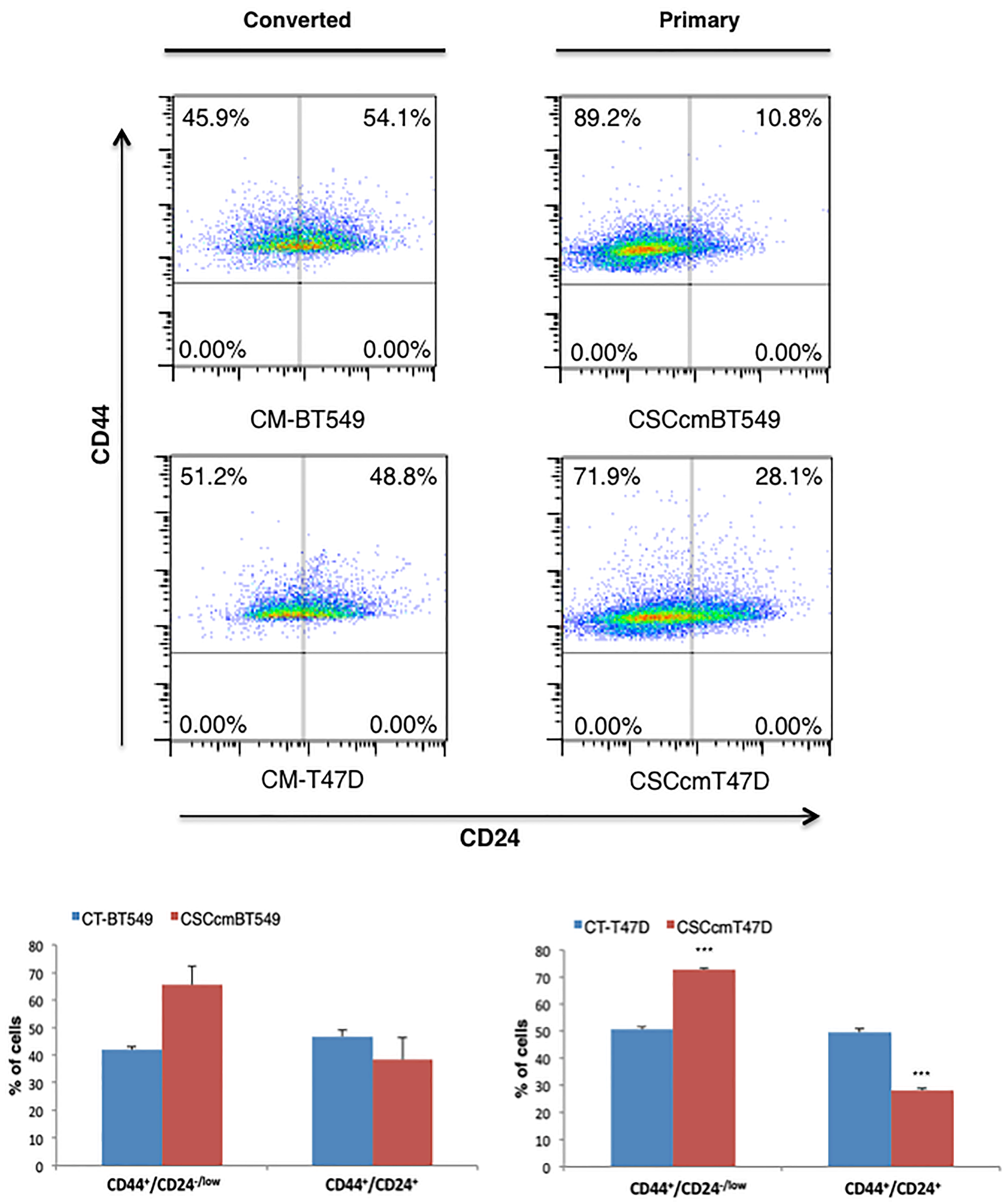

Supplementary Fig 2: Representative plots and corresponding graphs of flow cytometer analysis demonstrating percentage of CD24$^{-/low}$ and CD24^{+} in CD44^{+} cells in 1) CT-BT549, 2) CSCcmBT549, 3) CT-T47D and 4) CSCcmT47D. Flow cytometer plots are representative of three independent experiments. Data is presented as mean ± standard deviation and analyzed using unpaired two-tailed student's *t*-test. The level of significance was set as ***P<0.001, **P<0.01

Supplementary figure 3

a

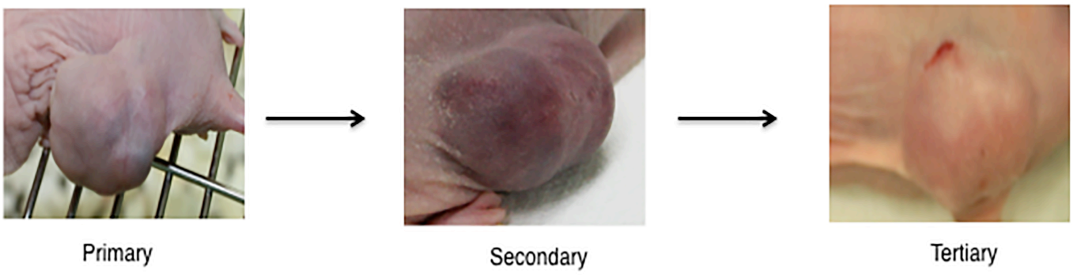

b

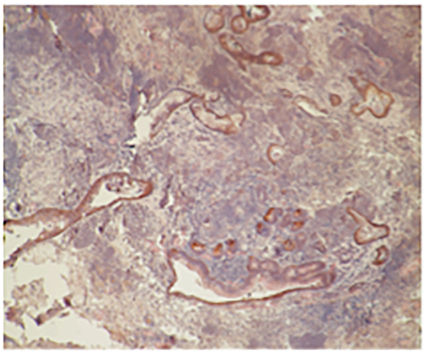

CSCcmBT549 injected into third mammary gland of Balb/c nude mice

Supplementary Fig 3: (a) Representative photographic images of serial subcutaneous tumors formed from CSCcmT47D cells in balb/c nude mice (b) Micrograph of primary orthotropic mammary tumor section formed by CSCcmBT549 stained with Her 2 receptor antibody showing Her2 overexpression. Scale bar represents 300µm.

Supplementary Figure 4

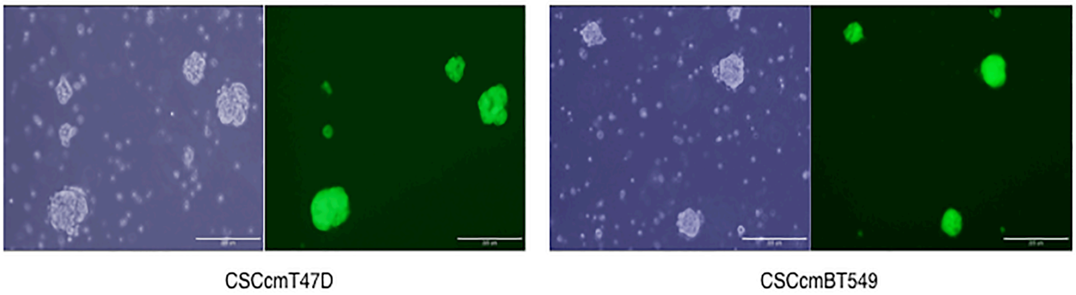

Supplementary Fig 4: Representative images of secondary spheres formed by CSCcmT47D and CSCcmBT549 primary spheres dissociated and cultured in serum free medium. Unstained cells in phase contrast and GFP-labeled cells in fluorescence contrast from each identical viewfield are shown side by side. Scale bars represent 200µm

Supplementary Figure 5

a

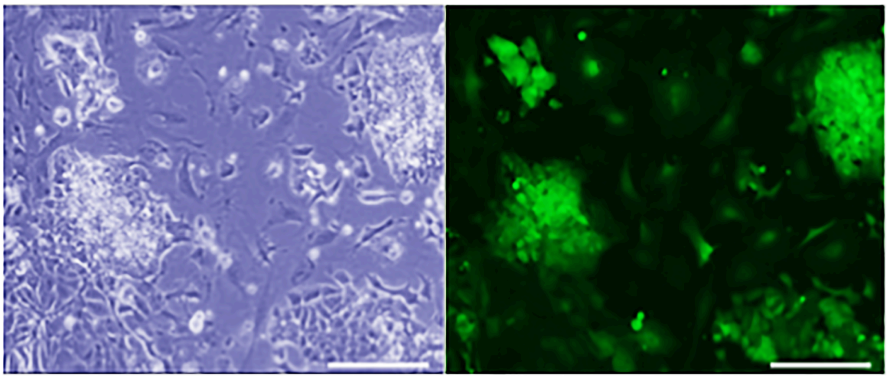

mESBT549 primary culture

b

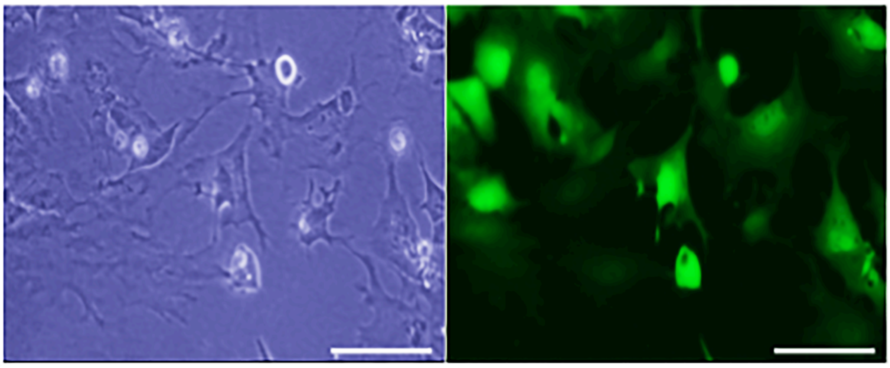

MACS separated fibroblast-like cells

Supplementary Fig 5: (a) Representative images of mESBT549 primary cells cultured from excised tumors. Scale bars represent 200µm (b) Representative images of fibroblasts separated from primary culture of mESBT549 using fibroblast specific magnetic activated cell sorting (MACS). Scale bars represent 100µm. Unstained cells in phase contrast and GFP-labeled cells in fluorescence contrast from each identical viewfield are shown side by side.

Supplementary Figure 6

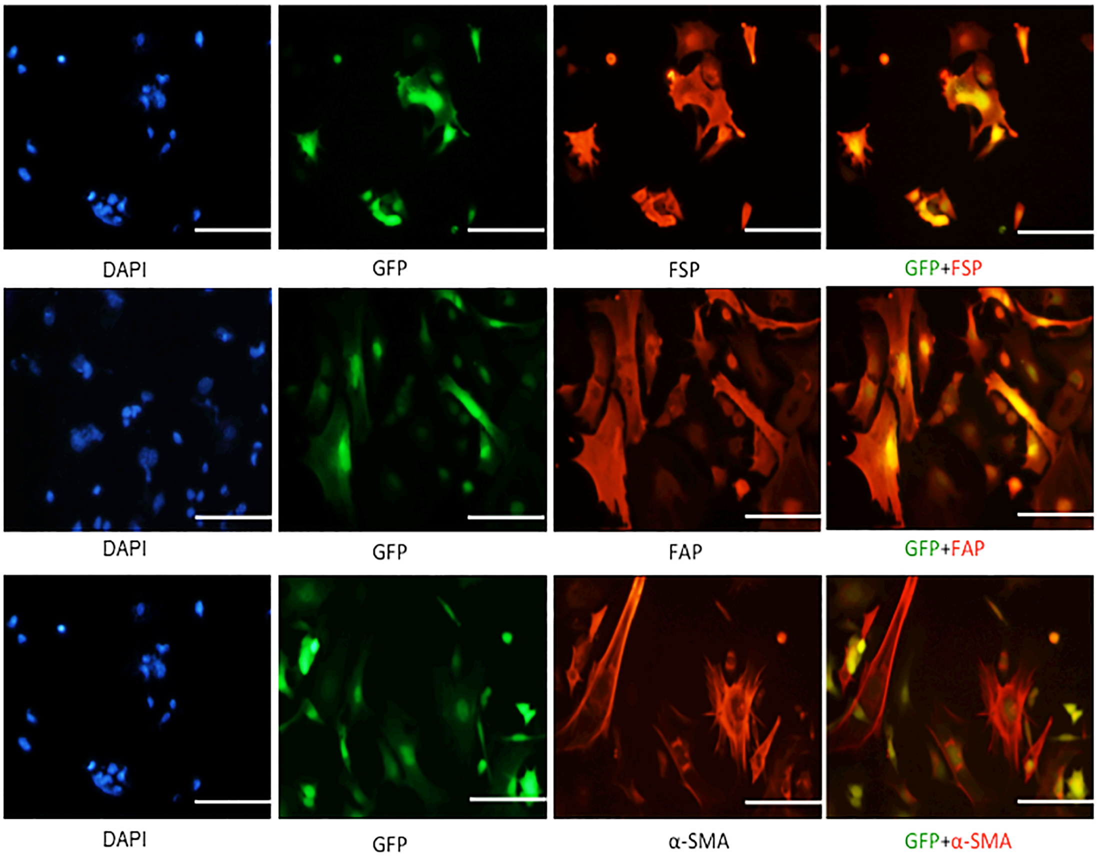

Supplementary Fig 6: MACS separated fibroblast-like cells were stained separately with CAF markers: anti-rabbit FSP1, anti-rabbit α-SMA and anti-rabbit FAP followed by staining with secondary antibodies: Alexa Fluor 555 and Alexa Fluor 488. The nuclei were counterstained with DAPI (*blue*). Scale bars represent 50μm.

Supplementary Figure 7

a

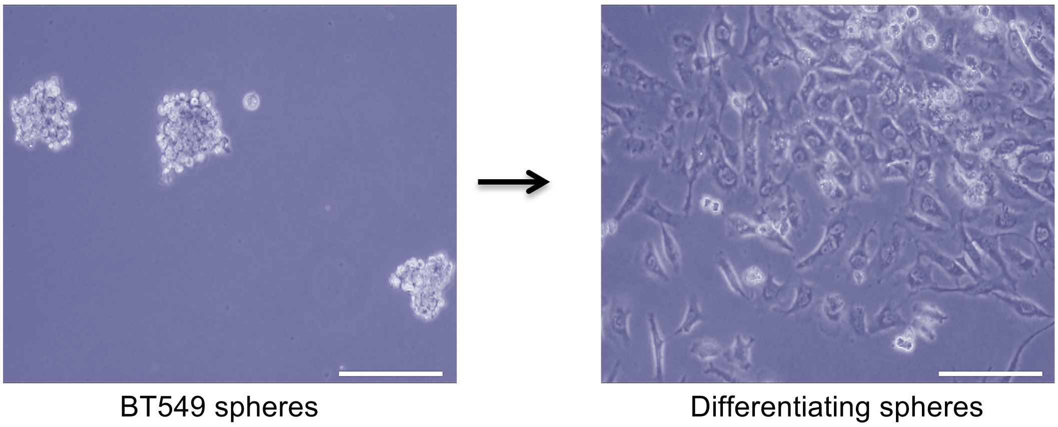

b

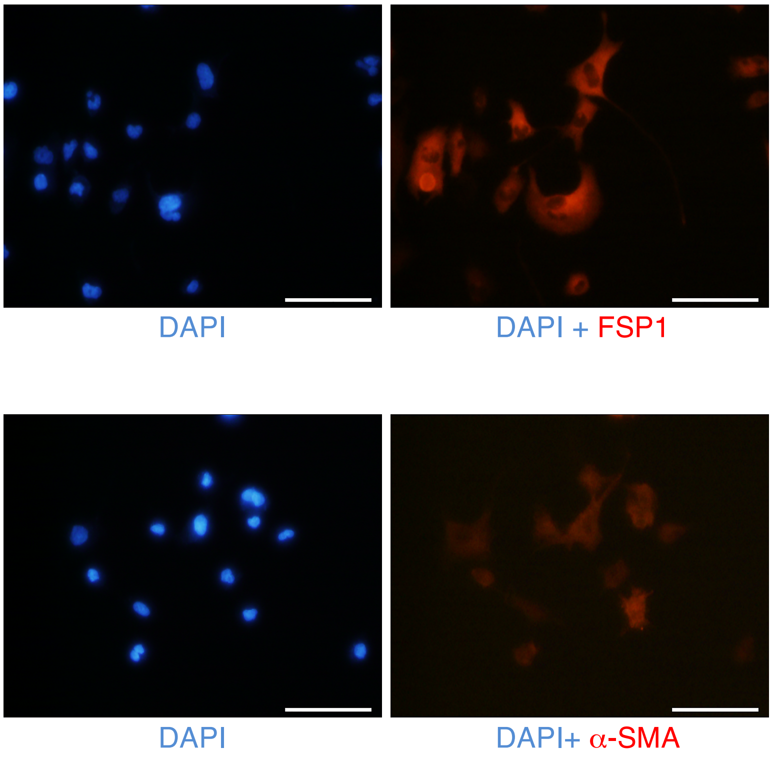

Supplementary Fig 7: (a) Representative brightfield images of breast cancer cell line, BT549 spheres and cells differentiated from spheres in presence of BT-549 conditioned medium collected in low serum. Scale bars represent 200µm. (b) Cells differentiated from spheres were stained separately with anti-rabbit FSP1 and anti-rabbit α-SMA primary antibodies. Scale bars represent 200µm.

Supplementary figure 8

a

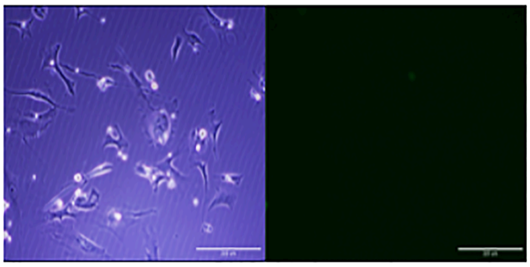

CSCcmT47D-CAFLC

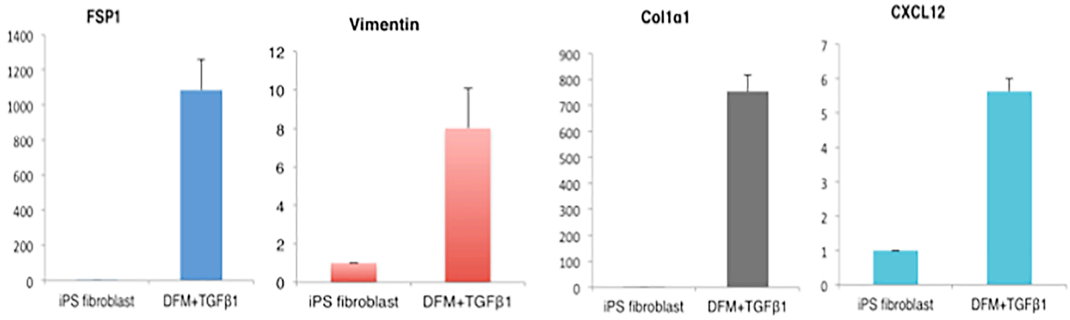

b

Supplementary Fig. 8: (a) Unstained cells showing morphology of cells differentiating from CSCcmT47D sphere treated with 10ng/ml of pure TGFβ1 growth factor in DMEM medium containing knock serum replacement medium. Phase contrast and fluorescence contrast images from identical viewfield were shown side by side. Scale bars represent 200μm (b) RT-qPCR analysis for CAF markers namely FSP1, Vimentin, Col1α1 and CXCL12 in CSCcmT47D-CAFLc in comparison with miPS-fibroblast.

Supplementary figure 9

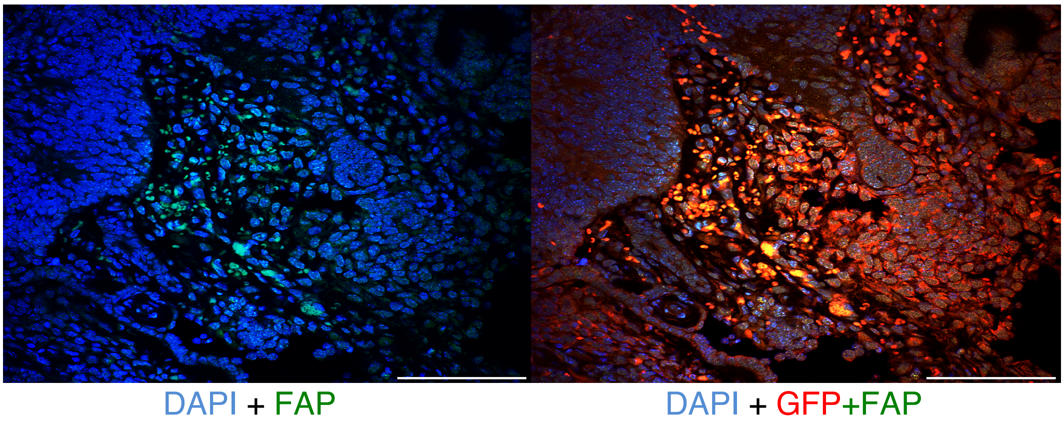

Supplementary Fig. 9: Representative images of Immunoflourescent stained CSCcmT47D sphere tumor with anti-rabbit fibroblast activation protein (Alexa fluor 488 secondary antibody-green), green fluorescent protein (Alexa fluor 555 conjugated with GFP antibody-red) antibodies. Nuclei were counterstained with DAPI (blue). Scale bars represent 50µm

Supplementary figure 10

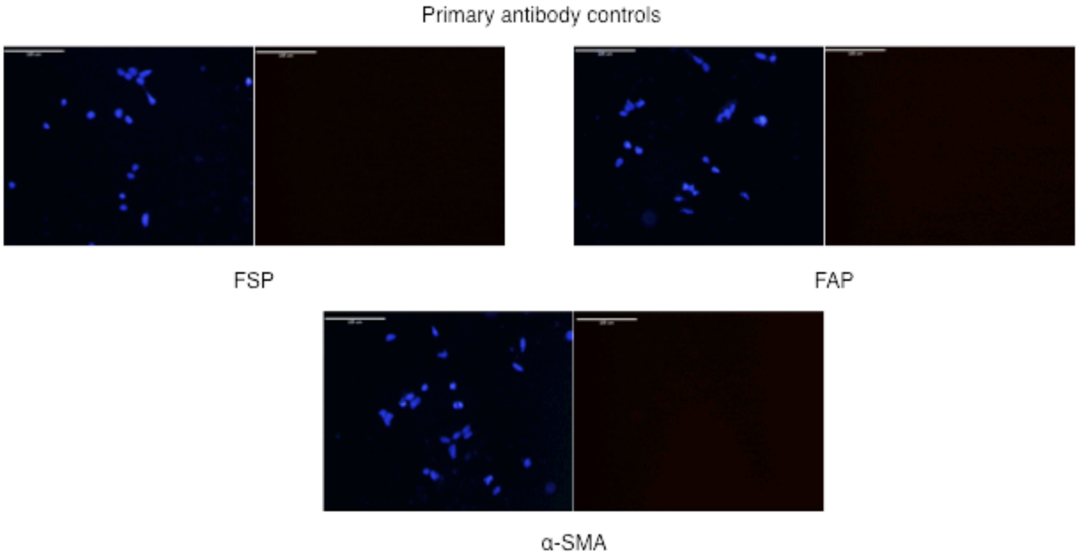

Supplementary Fig 10: Immunofluorescent analysis of CSCcmT47Dcm and CSCcmBT549 sphere differentiated CAF-like cells for primary antibody controls. Scale bars represent 100µm.

Supplementary Figure 11

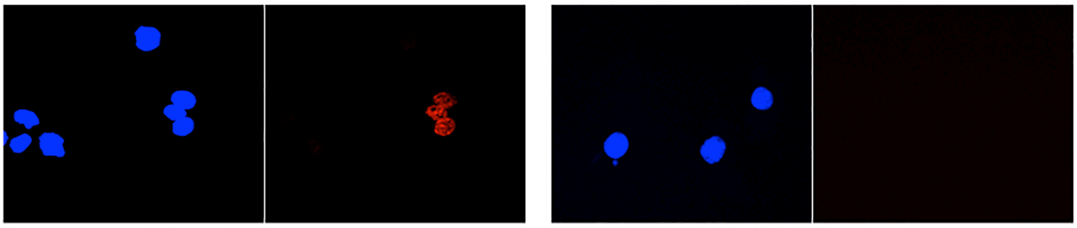

+Control – CSCcmT47D unseparated cells differentiating CSC

-Control – NIH3T3

Supplementary Fig 11: FISH assay positive control (sphere differentiated cells of CSCcmT47D before fibroblast separation) and negative control (NIH3T3). Scale bars represent 10μm.

Supplementary Figure 12

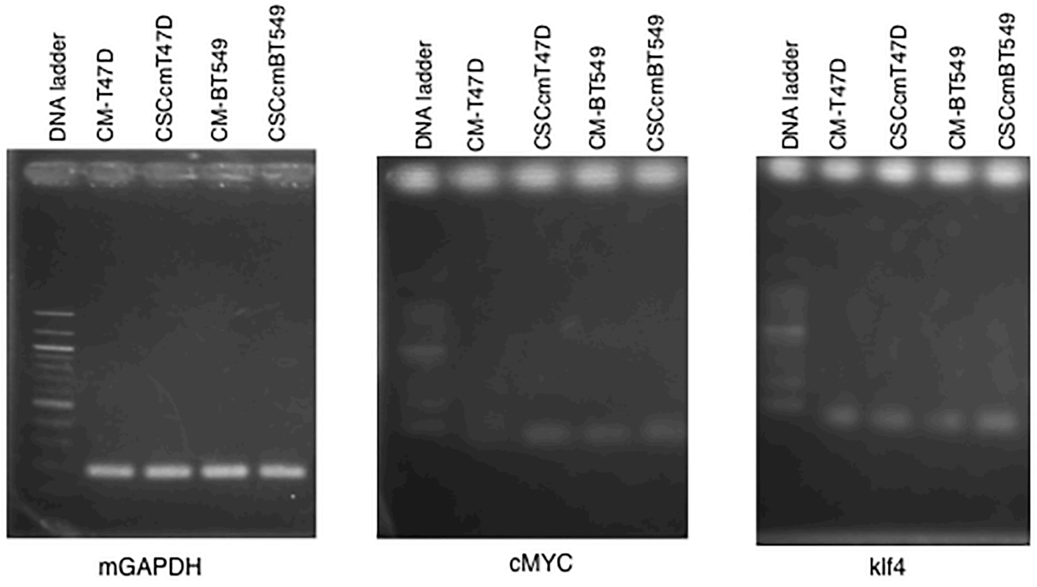

Supplementary Fig. 12: Agarose gel images showing amplification products for house keeping gene GAPDH, Tg-cMYC and Tg-klf4 in CM treated miPS cells (CM-T47D, CM-BT549) after four weeks and primary cells; CSCcmT47D and CSCcmBT549 generated from subcutaneous tumors.

Supplementary Table 1

List of mouse qPCR primers used in this study.

Gene	Forward Primer (5'-3')	Reverse Primer (5'-3')
CD133	CCTTGTGGTTCTTACGTTTGTTG	CGTTGACGACATTCTCAAGCTG
EpCam	CTGGCGTCTAAATGCTTGGC	CCTTGTCGGTTCTTCGGACTC
Oct3/4	TCTTTCCACCAGGCCCCCGGCTC	TGCGGGCGGACATGGGGAGATCC
Sox2	TAGAGCTAGACTCCGGGCGATGA	TTGCCTTAAACAAGACCACGAAA
Tg klf4	GCGAACTCACACAGGCGAGAAACC	TTATCGTCGACCACTGTGCTGCTG
Tg c-Myc	CAGAGGAGGAACGAGCTGAAGCGC	TTATCGTCGACCACTGTGCTGCTG
FSP1	CAGGCAAAGAGGGTGACAAG	TGCAGGACAGGAAGACACAG
α SMA	GGAGAAGCCCAGCCAGTCGC	AGCCGGCCTTACAGAGCCCA
Vimentin	CGGCTGCGAGAGAAATTGC	CCACTTTCCGTTCAAGGTCAAG
Col1α1	GTCCTAGTCGATGGCTGCTC	CAATGTCCAGAGGTGCAATG
PDGFα	ATGAGAGTGAGATCGAAGGCA	CGGCAAGGTATGATGGCAGAG
TGFβ1	TGATACGCCTGAGTGGCTGTCT	CACAAGAGCAGTGAGCGCTGAA
CXCL12	GAGCCAACGTCAAGCATCTG	CGGGTCAATGCACACTTGTC
